# Substituting Carbohydrate at Lunch for Added Protein Increases Fat Oxidation During Subsequent Exercise in Healthy Males

**DOI:** 10.1210/clinem/dgae237

**Published:** 2024-04-13

**Authors:** Tommy Slater, William J A Mode, Louise C Bonnard, Cian Sweeney, Mark P Funnell, Harry A Smith, John Hough, Ruth M James, Ian Varley, Craig Sale, James A Betts, Lewis J James, David J Clayton

**Affiliations:** Musculoskeletal Physiology Research Group, Sport, Health and Performance Enhancement Research Centre, School of Science and Technology, Nottingham Trent University, Nottingham, NG11 8NS, UK; National Institute for Health Research (NIHR) Leicester Biomedical Research Centre, Leicester, LE5 4PW, UK; Musculoskeletal Physiology Research Group, Sport, Health and Performance Enhancement Research Centre, School of Science and Technology, Nottingham Trent University, Nottingham, NG11 8NS, UK; Musculoskeletal Physiology Research Group, Sport, Health and Performance Enhancement Research Centre, School of Science and Technology, Nottingham Trent University, Nottingham, NG11 8NS, UK; Musculoskeletal Physiology Research Group, Sport, Health and Performance Enhancement Research Centre, School of Science and Technology, Nottingham Trent University, Nottingham, NG11 8NS, UK; National Centre for Sport and Exercise Medicine, School of Sport, Exercise and Health Sciences, Loughborough University, Loughborough, Leicestershire, LE11 3TU, UK; Department for Health, Centre for Nutrition Exercise and Metabolism, University of Bath, Bath, BA2 7AY, UK; Musculoskeletal Physiology Research Group, Sport, Health and Performance Enhancement Research Centre, School of Science and Technology, Nottingham Trent University, Nottingham, NG11 8NS, UK; Musculoskeletal Physiology Research Group, Sport, Health and Performance Enhancement Research Centre, School of Science and Technology, Nottingham Trent University, Nottingham, NG11 8NS, UK; Musculoskeletal Physiology Research Group, Sport, Health and Performance Enhancement Research Centre, School of Science and Technology, Nottingham Trent University, Nottingham, NG11 8NS, UK; Department of Sport and Exercise Sciences, Manchester Metropolitan University Institute of Sport, Manchester, M1 7EL, UK; Department for Health, Centre for Nutrition Exercise and Metabolism, University of Bath, Bath, BA2 7AY, UK; National Centre for Sport and Exercise Medicine, School of Sport, Exercise and Health Sciences, Loughborough University, Loughborough, Leicestershire, LE11 3TU, UK; Musculoskeletal Physiology Research Group, Sport, Health and Performance Enhancement Research Centre, School of Science and Technology, Nottingham Trent University, Nottingham, NG11 8NS, UK

**Keywords:** carbohydrate restriction, protein, fasting, exercise metabolism, appetite hormones, energy intake

## Abstract

**Context:**

How pre-exercise meal composition influences metabolic and health responses to exercise later in the day is currently unclear.

**Objective:**

Examine the effects of substituting carbohydrate for protein at lunch on subsequent exercise metabolism, appetite, and energy intake.

**Methods:**

Twelve healthy males completed 3 trials in randomized, counterbalanced order. Following a standardized breakfast (779 ± 66 kcal; ∼08:15), participants consumed a lunch (1186 ± 140 kcal; ∼13:15) containing either 0.2 g·kg^−1^ carbohydrate and ∼2 g·kg^−1^ protein (LO-CARB), or 2 g·kg^−1^ carbohydrate and ∼0.4 g·kg^−1^ protein (HI-CARB), or they fasted (FAST). Participants later cycled at ∼60% V̇O_2peak_ for 1 hour (∼16:15) and post-exercise ad libitum energy intake was measured (∼18:30). Substrate oxidation, subjective appetite, and plasma concentrations of glucose, insulin, nonesterified fatty acids (NEFA), peptide YY (PYY), glucagon-like peptide 1 (GLP-1), and acylated ghrelin were measured for 5 hours post-lunch.

**Results:**

Fat oxidation was greater during FAST (+11.66 ± 6.63 g) and LO-CARB (+8.00 ± 3.83 g) than HI-CARB (*P* < .001), with FAST greater than LO-CARB (+3.67 ± 5.07 g; *P* < .05). NEFA were lowest in HI-CARB and highest in FAST, with insulin demonstrating the inverse response (all *P* < .01). PYY and GLP-1 demonstrated a stepwise pattern, with LO-CARB greatest and FAST lowest (all *P* < .01). Acylated ghrelin was lower during HI-CARB and LO-CARB vs FAST (*P* < .01). Energy intake in LO-CARB was lower than FAST (−383 ± 233 kcal; *P* < .001) and HI-CARB (−313 ± 284 kcal; *P* < .001).

**Conclusion:**

Substituting carbohydrate for protein in a pre-exercise lunch increased fat oxidation, suppressed subjective and hormonal appetite, and reduced post-exercise energy intake.

Regular physical exercise is associated with numerous well-established health benefits (1) and can aid weight management (2). Interestingly, pre-exercise nutritional state can mediate the benefits of exercise, as performing a single bout of exercise after a prolonged fast (> 12 hours) has been shown to increase fat oxidation (3, 4) which, if performed regularly over a 6-week period, can improve fat oxidative capacity (5, 6). This is associated with improved markers of metabolic health (7), meaning regular overnight-fasted exercise training may augment improvements in insulin sensitivity (6, 8). Compared to fed exercise, overnight-fasted exercise may also aid in regulating energy balance. For example, compared to consuming breakfast before exercise, an acute bout of fasted exercise has no effect (4, 9, 10), or only slightly increases (3) lunch energy intake. This leads to a reduced cumulative energy intake which appears to persist over 24 hours (9), without any compensatory effects on 24-hour energy expenditure (3).

Most fasted exercise studies have been conducted in the morning, but morning exercise may not be possible or desirable for many. Macronutrient metabolism and appetite demonstrate circadian variation (11), so findings from overnight-fasted morning exercise may not translate to other times of day. One study showed that fasted exercise later in the day upregulates fat oxidation (12), but prolonged daytime fasting also elevates appetite, increases energy intake, and ultimately reduces motivation to exercise, exercise enjoyment, and exercise performance (13).

The metabolic benefits of fasted exercise may be driven by carbohydrate restriction, rather than fasting *per se*, with studies demonstrating that low-carbohydrate, high-protein feeding before morning exercise does not blunt fat oxidation compared to fasted exercise (14–16). Moreover, markers of training adaptation with implications for improved insulin sensitivity, such as AMPK signaling, as well as CD36 and PGC-1α mRNA expression, are upregulated following protein-only feeding (16–18). A high-protein meal also reduces appetite and energy intake to a greater extent than high-carbohydrate or high-fat meals (19), which may aid weight management efforts. This provides a practical rationale for overcoming some of the difficulties associated with conducting fasted exercise later in the day, but the metabolic and appetite-related effects of a low-carbohydrate, high-protein pre-exercise meal, relative to a more typical high-carbohydrate pre-exercise meal and fasting, are not well understood.

Exercise often takes place later in the day, meaning that the composition of meals consumed earlier in the day likely influence metabolic and health outcomes. However, the physiological and subjective responses to different pre-exercise meal compositions are not well understood. Therefore, the aim of this study was to examine the effects of consuming a low-carbohydrate, high-protein lunch prior to late-afternoon/early-evening (ie, 16:15) cycling exercise on substrate oxidation, compared with a high-carbohydrate, lower-protein lunch, or fasting. Secondary aims were to assess the effects of pre-exercise meal composition on exercise metabolism, appetite, and subsequent energy intake.

## Methods

### Participants

After ethical approval (Nottingham Trent University Ethical Advisory Committee: application number 704; ClinicalTrials registration no: NCT05107583), 12 healthy males completed the study (age 25 ± 2 years; height 1.81 ± 0.08 m; body mass 81.4 ± 10.2 kg; body fat 17 ± 6%; V̇O_2peak_ 45 ± 7 mL·kg^−1^·min^−1^). Participants were required to be aged 18 to 40 years, recreationally active (1-10 hours·wk^−1^), and self-reported to have had stable body weight for the 6 months before commencing the study to be included. Participants were excluded if they currently smoked, were classified as clinically restrained, disinhibited, or hungry eaters (20), had a severe dislike or intolerance of any study foods or drinks, were currently undergoing a structured diet and/or exercise intervention aiming to achieve weight loss, reported a history of gastric, digestive, cardiovascular, or renal disease, were taking medication or undergoing treatment known to affect glucose/lipid metabolism or appetite, or were consuming > 14 units of alcohol per week. Participants provided written informed consent and completed a health screening questionnaire before commencing the study.

Sample size was estimated using G*Power software (v3.1), an α of 0.05, and β of 0.90. Using fat oxidation data from a similar study, which observed an effect size of 0.54 when comparing carbohydrate- vs protein-fed exercise (15), it was estimated that 11 participants would be required to detect a 15% difference in fat oxidation during exercise. For energy intake, an energy deficit of 100 kcal·day^−1^ is recommended to prevent excess weight gain in 90% of the US population (21). Mean energy intake based on our previous work using an identical meal was ∼1070 kcal for males (13). Therefore, we deemed 10% a clinically meaningful difference between experimental conditions. Based on these values and an effect size of 0.61 from our previous study (13), it was estimated that 8 participants would be required to detect a 10% difference in post-exercise energy intake. As such, we recruited 12 participants to adequately power both aims of the study and enable a counterbalanced study design.

### Study Design

**Figure 1.**
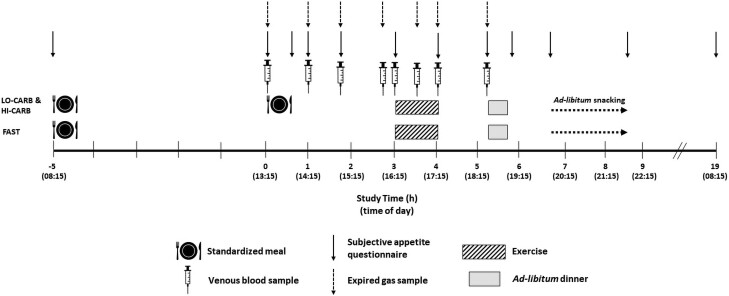
Schematic representation of the study protocol.

Participants completed 2 preliminary trials, and 3 experimental trials (completed between November 2021 and March 2022 in Nottingham Trent University laboratories) in a randomized (trial order drawn out of a bag), counterbalanced order, separated by ≥ 7 days (11 ± 7 days). Experimental trials involved consuming a standardized carbohydrate-rich breakfast at home (779 ± 66 kcal; ∼08:15), before a lunch (1186 ± 140 kcal; ∼13:15) containing either 0.2 g·kg body mass^−1^ carbohydrate and ∼2 g·kg body mass^−1^ protein (LO-CARB), 2 g·kg body mass^−1^ carbohydrate and ∼0.4 g·kg body mass^−1^ protein (HI-CARB), or a water-only lunch (FAST) in the laboratory. The energy content of the LO-CARB and HI-CARB meals was matched by substituting carbohydrate for protein, while keeping fat content similar. Three hours later (∼16:15), participants completed 1 hour of cycling (∼60% V̇O_2peak_), before ad libitum energy intake was assessed at dinner and from a selection of snacks provided after participants left the laboratory (Fig. 1). Participants were blinded to the compositional differences between LO-CARB and HI-CARB meals until completion of the study. Trials took place in a laboratory (20.6 ± 3.0 °C, 44.9 ± 8.7% relative humidity, 751 ± 8 mmHg barometric pressure).

### Preliminary Trials

The first preliminary trial involved measuring participants’ body mass, height, and skinfold thickness (22), before V̇O_2peak_ was determined on an electronically braked cycle ergometer (Lode Corival, Netherlands). The test involved 4-minute incremental stages separated by ∼5 minutes rest until volitional exhaustion, with 1-minute expired gas samples collected during the final minute of each increment. The second preliminary trial involved familiarization with key aspects of the experimental protocol (ie, cycling and ad libitum eating procedures).

### Pretrial Standardization

Participants recorded all dietary intake (including caffeine intake) and physical activity in the 24 hours before the first experimental trial, replicating this before subsequent trials. Strenuous activities and alcohol intake were prohibited during this period, with adherence confirmed verbally upon arrival at the laboratory prior to each trial. On the evening before trials, participants ceased food and caffeine intake at 20:00 and fasted overnight (other than plain water, which was standardized between trials). Participants arrived at the laboratory via motorized transport.

### Protocol

At ∼08:15 (−5 hours), participants completed baseline measures of subjective appetite before consuming a standardized breakfast. Participants arrived at the laboratory between 12:15-12:45 (consistent between trials for each participant). An indwelling cannula was inserted into an antecubital vein, and after 30 minutes of supine rest, a baseline blood sample, expired gas sample, and subjective appetite measures, were collected. At ∼13:15 (0 hours), participants consumed an experimental lunch meal (LO-CARB and HI-CARB), or volume of water equal to the water content of LO-CARB and HI-CARB meals (FAST). After lunch (0.5 hours), subjective perceptions of the meal were collected (LO-CARB and HI-CARB only), and participants rested in the laboratory, with blood and expired gas samples collected after 30 minutes of supine rest at 1, 1.75, and 2.75 hours. At 3 hours (∼16:15), subjective measures of appetite, mood, exercise readiness, and a pre-exercise blood sample were collected before participants completed 1 hour of cycling at an intensity calculated to elicit ∼60% V̇O_2peak_. During exercise, expired gas samples were collected between 28 to 30 and 58 to 60 minutes, venous blood samples collected at 30 and 60 minutes, and heart rate and rating of perceived exertion recorded every 15 minutes, with subjective appetite and exercise enjoyment measured immediately post-exercise. Final expired gas and venous blood samples were collected 1 hour post-exercise after 30 minutes of supine rest. An ad libitum meal was served 1.25 hours post-exercise (∼18:30), with participants permitted 20 minutes to eat. Participants then left the laboratory, taking a selection of snacks which they could choose to consume ad libitum at home between 20:00 and 22:00 only. Outside of the snacking window, participants were instructed to refrain from food and caffeine intake until after the final subjective appetite questionnaire was completed at 08:15 the following day, although ad libitum water intake was permitted (volume recorded). Adherence to these instructions was confirmed via text messaging.

### Standardized Breakfast Meal

Meals were provided as a percentage of estimated energy requirements (EER; resting metabolic rate (23) multiplied by a physical activity factor of 1.7 to account for the exercise component of the trial). A standardized breakfast (25% EER; Table 1) consisting of porridge (Oatso Simple Golden Syrup, Quaker, UK), cereal bars (Belvita, Mondelez, UK), yogurt (Ski Strawberry, Nestlé, UK), and strawberry milkshake (Yazoo, Campina Ltd., UK) was provided in all experimental trials (Supplementary Table S1) (24).

**Table 1.** Macronutrient composition of each meal

	Carbohydrate (g)	Protein (g)	Fat (g)	Fiber (g)	Energy (kcal)
**Standardized breakfast**					
All	121.2 ± 9.2	24.1 ± 1.9	20.0 ± 1.7	9.4 ± 0.7	779 ± 66
Experimental lunch					
LO-CARB	18.4 ± 2.5	157.7 ± 18.8	50.1 ± 5.9	15.3 ± 1.4	1186 ± 140*^b^*
HI-CARB	163.2 ± 19.3	30.6 ± 3.9	44.3 ± 5.3	6.0 ± 0.5	1186 ± 140*^c^*
FAST	0	0	0	0	0*^bc^*
Ad libitum dinner					
LO-CARB	149.8 ± 38.0	23.6 ± 6.0	17.6 ± 4.5	8.2 ± 2.1	869 ± 220*^ab^*
HI-CARB	186.9 ± 43.1	29.4 ± 6.8	22.0 ± 5.1	10.3 ± 2.4	1084 ± 250*^a^*
FAST	195.0 ± 50.3	30.7 ± 7.9	22.9 ± 5.9	10.7 ± 2.8	1131 ± 292*^b^*
Ad libitum snack					
LO-CARB	83.1 ± 39.3	7.2 ± 3.5	22.9 ± 11.4	4.0 ± 2.9	575 ± 272
HI-CARB	98.8 ± 40.2	9.0 ± 4.4	25.5 ± 7.7	6.4 ± 3.9	673 ± 245
FAST	101.7 ± 37.7	9.6 ± 3.1	26.5 ± 9.6	6.2 ± 3.1	696 ± 246
**Total**					
LO-CARB	372.5 ± 60.4	212.6 ± 24.8	110.6 ± 16.0	36.8 ± 5.2	3409 ± 466*^ab^*
HI-CARB	570.1 ± 78.8	93.1 ± 11.4	111.7 ± 13.0	32.0 ± 5.6	3722 ± 478*^ac^*
FAST	417.8 ± 67.8	64.4 ± 9.8	69.4 ± 11.1	26.3 ± 4.9	2606 ± 403*^bc^*

Data are mean ± SD. Abbreviations: FAST, no lunch (extended fasting) experimental trial; HI-CARB: high-carbohydrate lunch experimental trial; LO-CARB, low-carbohydrate lunch experimental trial.

^*a*^LO-CARB vs HI-CARB total energy intake (*P* < .05).

^*b*^LO-CARB vs FAST total energy intake (*P* < .05).

^*c*^HI-CARB vs FAST total energy intake (*P* < .05).

### Experimental Lunch Meals

In HI-CARB and LO-CARB, lunch (35%-40% EER) consisted of tuna and mayonnaise sandwiches, crisps, and a blended drink. Meals provided either 0.2 g·kg body mass^−1^ carbohydrate and ∼2.0 g·kg body mass^−1^ protein (LO-CARB) or 2 g·kg body mass^−1^ carbohydrate and ∼0.4 g·kg body mass^−1^ protein (HI-CARB) (Table 1). The energy content of meals was matched primarily via the manipulation of carbohydrate and protein content. Fat content was closely matched between trials to limit effects of dietary fat on substrate oxidation (25). The bread (Hovis, UK) and crisps (Walkers, UK) provided in HI-CARB were substituted for low-carbohydrate, high-protein bread (LivLife, UK) and crisps (MyProtein, UK) in LO-CARB. The HI-CARB blended drink consisted of water, maltodextrin (MyProtein, UK), full-fat milk, chocolate milkshake powder (Nesquik, Nestlé, UK), sucralose sweetener (ASDA, UK), and thickening agent xanthan gum (Doves Farm, UK). The LO-CARB drink consisted of water, chocolate-flavored soy protein isolate (MyProtein, UK), double cream (ASDA, UK), and sucralose sweetener (ASDA, UK) (Supplementary Table S1) (24). In FAST, participants consumed water equal to the water content of LO-CARB and HI-CARB meals. To eliminate the possibility of water intake influencing appetite, water intake was provided at 30 mL·kg body mass^−1^ in all trials, distributed into 7 equal volumes consumed: 08:15-10.30; 10:30-12:30; 14:15-15:15; 15:15-16:15; first half of exercise (16:15-16:45); second half of exercise (16:45-17:15); 17:15-18:15.

### Ad Libitum Energy Intake

Ad libitum dinner energy intake was determined by weighing food items before and after consumption. Dinner was a homogenous meal consisting of pasta, tomato sauce, and extra virgin olive oil, containing 1.25 ± 0.01 kcal·g^−1^ (69% carbohydrate, 11% protein, 18% fat, and 2% fiber), and was provided in excess of expected consumption (Supplementary Table S2) (24). Participants ate in isolation to eliminate distractions until they felt *“*comfortably full and satisfied,” with water available ad libitum. Participants remained in the booth for the entire 20-minute period and reported ceasing to eat within this time in all trials. In all experimental trials, the selection of snacks which could be consumed ad libitum at home included 4 chocolate bars (Mars, UK), 2 cereal bars (Special K, Kellogg's, UK), 2 packets of ready salted crisps (Walkers, UK), 2 apples, and 2 satsumas (Supplementary Table S2) (24). All items consumed outside the laboratory were weighed before being provided and reweighed the following day within the laboratory after collection from the participant by a researcher. The energy densities used to derive total energy intake from each macronutrient were: 4 kcal·g^−1^ for carbohydrate, 4 kcal·g^−1^ for protein, 9 kcal·g^−1^ for fat, and 2 kcal·g^−1^ for fiber.

### Expired Gas Samples

At rest, expired gas was sampled for 10 minutes after the participant had lain supine for 25 minutes. The first 5 minutes served as a familiarization period, after which the sample was discarded. During the second 5 minutes, expired gas was collected into a Douglas bag for analysis. During exercise, expired gas was sampled for 3 minutes, which included a 1-minute familiarization period (sample discarded), with the subsequent 2 minutes sample collected and analyzed. Expired gas was analyzed for oxygen and carbon dioxide concentrations (MiniHF 5200, Servomex, UK), volume (Harvard Dry Gas Meter, Harvard Ltd., UK), and temperature, and substrate oxidation rates calculated (26).

### Subjective Responses

Participants rated hunger, fullness, desire to eat (DTE), prospective food consumption (PFC), and nausea on digital visual analog scales (VAS) sent to their mobile telephone at −5, 0, 0.5, 1, 1.75, 3, 4, 5.25, 5.75, 6.75, 8.75, and 19 hours (relative to lunch). Additionally, motivation to exercise, readiness to exercise, tiredness, and energy, were rated pre-exercise (3 hours). VAS were administered using Surveymonkey.com, with 0-100 sliding scales including written anchors “not at all”/“no desire at all”/“none at all” and “extremely”/“a lot” at 0 and 100, respectively. Participants also completed a paper-based Positive and Negative Affect Schedule (PANAS) (27) pre-exercise. Enjoyment of exercise was assessed immediately post-exercise using a paper-based, shortened version of the Physical Activity Enjoyment Scale (PACES-8) (28).

Additional VAS relating to perceptions of the overall meal (how pleasant), the sandwich (how pleasant, dry, moist, chewy), and the drink (how pleasant, bitter, sweet, creamy, thick, sticky, salty) were completed by participants immediately after lunch in LO-CARB and HI-CARB.

### Blood Sampling and Analyses

Blood samples (∼10 mL) were drawn from an antecubital vein. The first 2 mL was discarded, before 4.9 mL blood was collected into an EDTA monovette (1.6 mg·mL^−1^; Sarstedt AG & Co., Germany). A further 2.7 mL blood was collected into an EDTA monovette (1.6 mg·mL^−1^) containing 27 µL of a potassium phosphate buffer (PBS; 0.05 M), P-hydroxymercuribenzoic acid (PHMB; 0.05 M), and sodium hydroxide (NaOH; 0.006 M) solution, to prevent degradation of acylated ghrelin. Following collection, blood samples were centrifuged (1700*g*, 15 minutes, 4 °C), the supernatant (1 mL) of the PHMB/PBS/NaOH-treated blood was mixed with 100 μL hydrochloric acid (1 M), and plasma was stored at −80 °C until analysis. Acylated ghrelin (intra-assay coefficient of variation [CV] 1.8%-6.2%; limit of detection [LoD] <5 pg·mL^−1^; Bertin Technologies, France; Catalog #A05106, RRID: AB_3083805), insulin (intra-assay CV 2.7%-5.8%; LoD 6 pmol·L^−1^; Mercodia, Sweden; Catalog #10-1113-01, RRID: AB_2877672), total peptide YY (PYY) (intra-assay CV 1.6%-4.0%; LoD 6.5 pg·mL^−1^; Merck Millipore Ltd., UK; Catalog #EZHPYYT-66 K, RRID: AB_2910201), and total glucagon-like peptide 1 (GLP-1) (intra-assay CV 2.2%-4.4%; LoD 1.5 pM·L^−1^; Merck Millipore Ltd.; Catalog #EZGLP1T-36 K, RRID: AB_2813786) concentrations were determined by enzyme-linked immunosorbent assay (ELISA). Plasma glucose (intra-assay CV 0.2%-0.4%; LoD 0.1 mmol·L^−1^; Horiba Ltd., UK), nonesterified fatty acid (NEFA; intra-assay CV 1.0%; LoD 0.072 mmol·L^−1^; Randox Laboratories Ltd., UK), and glycerol (intra-assay CV 7.5%; LoD 14.5 μmol·L^−1^; Randox Laboratories Ltd.) concentrations were determined by enzymatic colorimetric assay. To avoid inter-assay variation from influencing results, samples from the same participant were analyzed within the same run/assay.

### Statistical Analyses

Data were analyzed using SPSS v26.0 (IBM, USA). Raw data were checked for normality using a Shapiro-Wilk test. Incremental area under the curve (iAUC) or total area under the curve (tAUC) were calculated with the trapezoid method using the Time Series Response Analyzer tool (29). For appetite-related variables, AUC values were determined in response to breakfast (08:15-13:15), lunch (13:15-16:15), exercise (16:15-18:30), and dinner/overnight (18:30-08:15). Data containing 1 factor (ad libitum energy intake, exercise subjective responses, laboratory conditions, iAUC, and tAUC) were analyzed using one-way repeated-measures analysis of variance (ANOVA) and data containing 2 factors (plasma substrate/hormone concentrations, energy expenditure and substrate oxidation rates, and subjective appetite sensations) were analyzed using two-way repeated-measures ANOVA. Assumptions of sphericity of the ANOVA were checked and adjustments for the degrees of freedom were made using the Greenhouse-Geiser (ε < 0.75) or Huynh-Feldt (ε > 0.75) correction, where appropriate. Significant ANOVA effects were explored with post hoc paired samples *t* tests (normally distributed data), or Wilcoxon Signed-Rank tests (non-normally distributed data), with Holm-Bonferroni stepwise correction. Data sets were considered statistically different when *P* < .05. Data are presented as mean ± SD, unless stated. Effect sizes (Cohen's *dz*) were calculated, with 0.2, 0.5, and 0.8 representing small, medium, and large effect sizes (30). A one-way repeated-measures ANOVA was conducted on the primary outcome of fat oxidation during exercise to show no systematic effect of trial order (*P ≤* .677).

## Results

### Energy Expenditure and Substrate Oxidation

**Figure 2.**
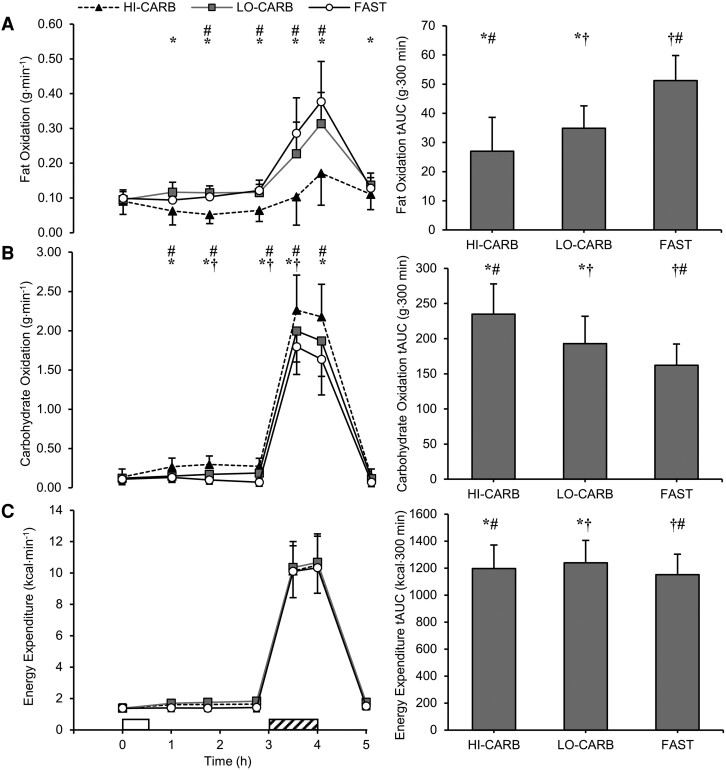
(A) Fat oxidation, (B) carbohydrate oxidation, and (C) energy expenditure during HI-CARB, LO-CARB, and FAST. Data are presented at each time point (left) and as total area under the curve (tAUC) for each trial (right). Data are mean ± SD. White rectangle represents standardized lunch; diagonal striped rectangle represents exercise. *LO-CARB vs HI-CARB (*P* < .05); †LO-CARB vs FAST (*P* < .05); #HI-CARB vs FAST (*P* < .05).

There were trial-by-time interaction effects for fat and carbohydrate oxidation rates (both *P* < .001), where total fat oxidation across the trial period was higher and carbohydrate oxidation lower in LO-CARB and FAST vs HI-CARB, as well as in FAST vs LO-CARB (*dz* = 0.98-2.03; all *P* < .01; see Fig. 2 for specific time points). There was a main effect of trial (*P* < .001), but no trial-by-time interaction effect for energy expenditure (*P* = .119). Total energy expenditure across the trial period was greater in LO-CARB (*dz* = 2.49; *P* < .001) and HI-CARB (*dz* = 0.87; *P* = .012) vs FAST, and in LO-CARB vs HI-CARB (*dz* = 1.00; *P* < .01; Fig. 2).

**Figure 3.**
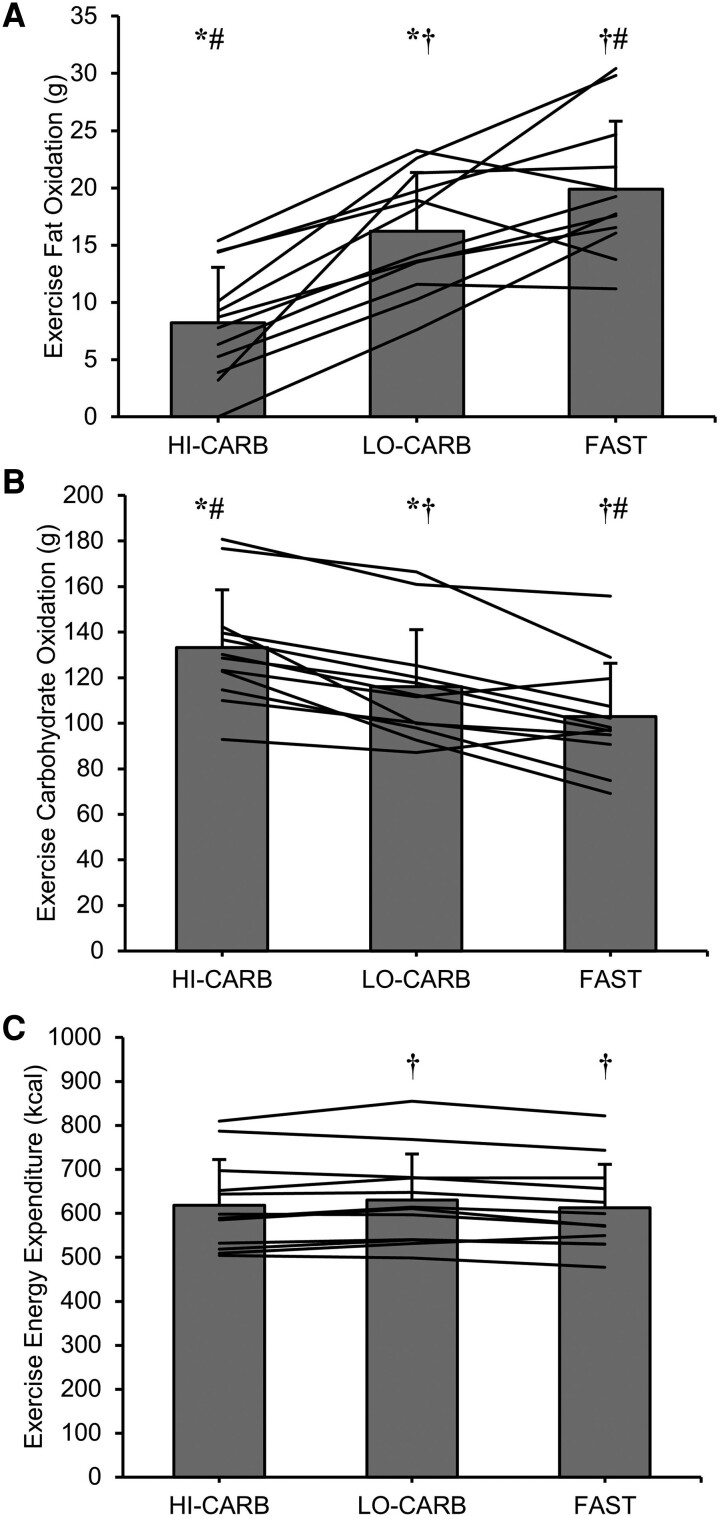
(A) Total fat oxidation, (B) total carbohydrate oxidation, and (C) total energy expenditure during the 1 hour cycling exercise in HI-CARB, LO-CARB, and FAST. The bars display mean values, with vertical error bars representing SD. The lines display individual subjects’ substrate oxidation and energy expenditure for each experimental trial. *LO-CARB vs HI-CARB (*P* < .05); †LO-CARB vs FAST (*P* < .05); #HI-CARB vs FAST (*P* < .05).

During exercise, fat oxidation was 8.00 ± 3.83 g greater in LO-CARB (*dz* = 2.10; *P* < .001) and 11.66 ± 6.63 g greater in FAST (*dz* = 1.75; *P* < .001) vs HI-CARB and was also 3.67 ± 5.07 g greater in FAST vs LO-CARB (*dz* = 0.73; *P* = .029). Carbohydrate oxidation was 17.21 ± 10.16 g lower in LO-CARB (*dz* = 1.71; *P* < .01) and 30.25 ± 17.39 g lower in FAST (*dz* = 1.75; *P* < .001) vs HI-CARB and was also 13.04 ± 13.55 g lower in FAST vs LO-CARB (*dz* = 0.98; *P* < .01). Exercise energy expenditure was 17 ± 16 kcal greater in LO-CARB vs FAST (*dz* = 1.12; *P* < .01; Fig. 3).

### Energy Intake

Ad libitum dinner energy intake in LO-CARB was 262 ± 174 kcal lower than FAST (*dz ­*= 1.52; *P* < .001) and 215 ± 135 kcal lower than HI-CARB (*dz* = 1.58; *P* < .001) but was not different between FAST and HI-CARB (*dz* = 0.41; *P* = .194; Table 1). Snack energy intake (LO-CARB: 575 ± 272 kcal, FAST: 696 ± 246 kcal, HI-CARB: 673 ± 245 kcal; *dz ­*= 0.09-0.50; *P* = .274) and macronutrient intake (*dz ­*= 0.05-0.84; all *P* ≥ .055) were not different between trials. Cumulative energy intake across the day was 803 ± 279 kcal greater in LO-CARB (*dz ­*= 2.86; *P* < .001) and 1116 ± 315 kcal greater in HI-CARB (*dz ­*= 3.56; *P* < .001) vs FAST but was also 313 ± 284 kcal greater during HI-CARB than LO-CARB (*dz ­*= 1.10; *P* < .01).

### Blood Parameters

**Figure 4.**
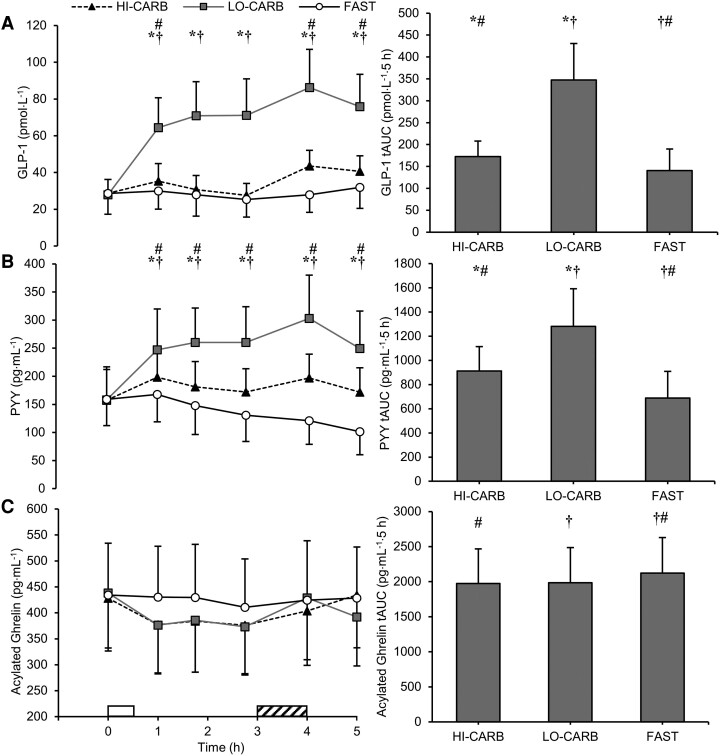
Plasma concentrations of (A) GLP-1, (B) PYY, and (C) acylated ghrelin during HI-CARB, LO-CARB, and FAST. Data are presented at each time point (left) and as total area under the curve (tAUC) for each trial (right). Data are mean ± SD (GLP-1 and PYY) or mean ± SEM (acylated ghrelin). White rectangle represents standardized lunch; diagonal striped rectangle represents exercise. *LO-CARB vs HI-CARB (*P* < .05); †LO-CARB vs FAST (*P* < .05); #HI-CARB vs FAST (*P* < .05).

There were trial-by-time interaction effects for plasma PYY and GLP-1 concentrations (both *P* < .001). For plasma acylated ghrelin concentrations, there was a main effect of trial (*P* < .001), but no trial-by-time interaction effect (*P* = .067). *­*PYY and GLP-1 tAUC were greater in LO-CARB (*dz* = 2.66-4.01; both*­ P* < .001) and HI-CARB (*dz* = 1.16-1.48; both*­ P* < .01) vs FAST and in LO-CARB vs HI-CARB (*dz* = 1.98-3.22; both *P ­*< .001; see Fig. 4 for specific time points). Acylated ghrelin tAUC was lower in LO-CARB (*dz* = 1.19; *P* < .01*­*) and HI-CARB (*dz* = 1.32; *P* < .01) vs FAST (Fig. 4).

**Figure 5.**
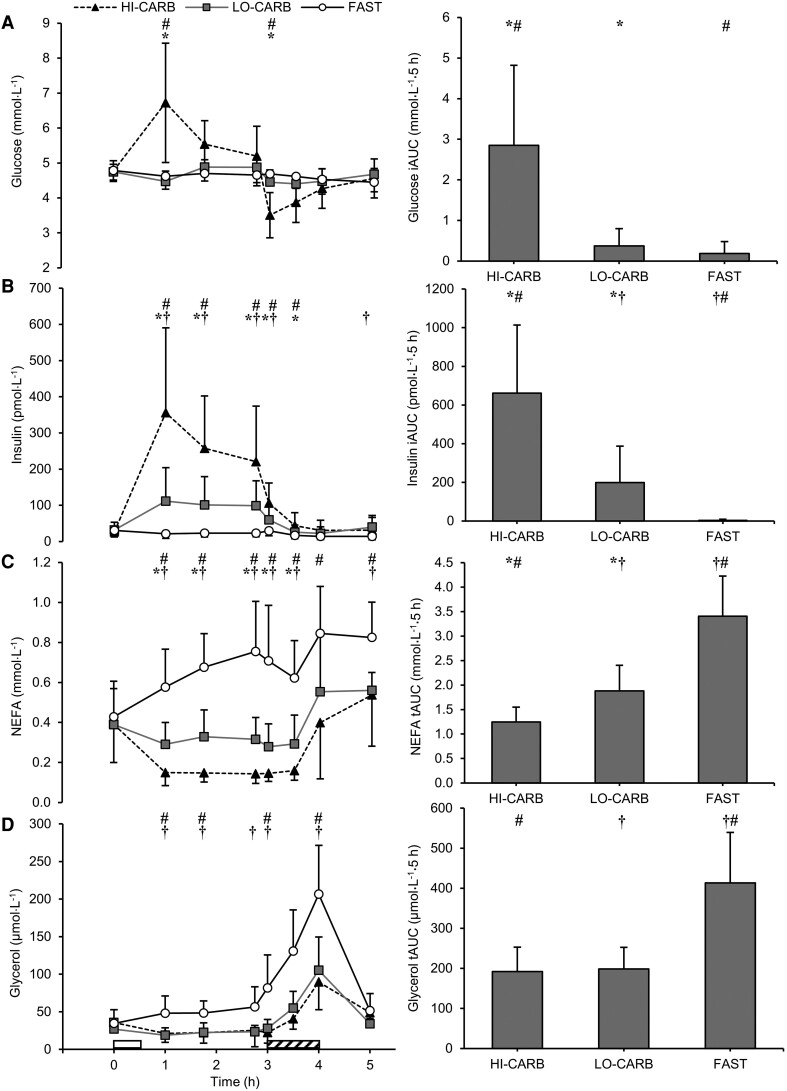
Plasma concentrations of (A) glucose, (B) insulin, (C) nonesterified fatty acids (NEFA), and (D) glycerol during HI-CARB, LO-CARB, and FAST. Data are presented at each time point (left) and as incremental area under the curve (iAUC) or total area under the curve (tAUC) for each trial (right). Data are mean ± SD. White rectangle represents standardized lunch; diagonal striped rectangle represents exercise. *LO-CARB vs HI-CARB (*P* < .05); †LO-CARB vs FAST (*P* < .05); #HI-CARB vs FAST (*P* < .05).

There were trial-by-time interaction effects for plasma insulin, glucose, NEFA, and glycerol concentrations (all *P* < .001). Insulin iAUC was lower in LO-CARB (*dz* = 1.65; *P* < .001) and FAST (*dz* = 1.87; *P* < .001) vs HI-CARB and in FAST vs LO-CARB (*dz ­*= 1.04; *P* < .01; see Fig. 5 for specific time points). Glucose iAUC was lower in both LO-CARB (*dz* = 1.17; *P* < .01) and FAST (*dz ­*= 1.41; *P* < .01) vs HI-CARB, although glucose was lower at 1 hour (*dz ­*= 1.16-1.31; both *P* ≤ .036), and higher at 3 hours (*dz ­*= 1.54-1.68; both *P* < .01), in LO-CARB and FAST vs HI-CARB. tAUC for NEFA was greater in LO-CARB (*dz* = 0.93; *P* < .01) and FAST (*dz* = 2.61; *P* < .001) vs HI-CARB, and in FAST vs LO-CARB (*dz* = 1.31; *P* < .01; see Fig. 5 for specific time points). tAUC for glycerol was greater in FAST vs LO-CARB (*dz* = 1.48; *P* < .01) and HI-CARB (*dz* = 1.78; *P* < .001; see Fig. 5 for specific time points).

### Subjective Appetite Responses

**Figure 6.**
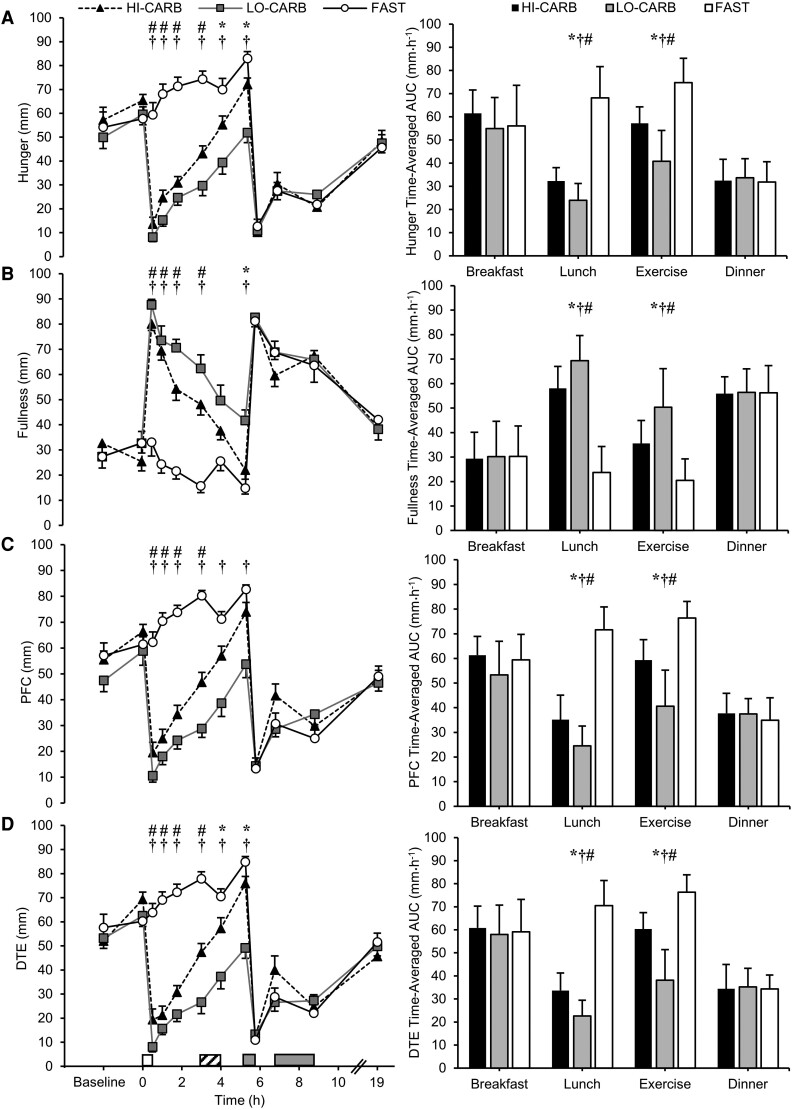
(A) Hunger, (B) fullness, (C) prospective food consumption (PFC), and (D) desire to eat (DTE) during HI-CARB, LO-CARB, and FAST. Data are presented at each time point (left) and as time-averaged total area under the curve (tAUC) for each trial (right). Data are mean ± SEM. White rectangle represents standardized lunch; gray rectangle represents ad libitum dinner and snacking; diagonal striped rectangle represents exercise. *LO-CARB vs HI-CARB (*P* < .05); †LO-CARB vs FAST (*P* < .05); #HI-CARB vs FAST (*P* < .05).

There were trial-by-time interaction effects for hunger, fullness, DTE, and PFC (all *P <* .001; Fig. 6), but not nausea (*P* = .367). Following lunch, values for hunger, DTE, and PFC were lower, and fullness was higher until 3 hours in LO-CARB and HI-CARB vs FAST (*dz* = 1.60-3.66; all *P* < .001), and these differences were still apparent at 4 (*dz ­*= 1.04-1.73; all *P* < .001) and 5.25 hours (*dz* = 1.43-2.00; all *P* < .001) between LO-CARB and FAST. Hunger and DTE were lower in LO-CARB vs HI-CARB at 4 and 5.25 hours, with fullness also higher at 5.25 hours in LO-CARB (*dz ­*= 1.30-1.56; all *P* ≤ .019).

Hunger, DTE, and PFC tAUC were all lower, and fullness tAUC greater in response to lunch and exercise in LO-CARB (*dz* = 1.75-3.55; all *P* < .001) and HI-CARB (*dz* = 1.47-2.87; all *P* < .01) vs FAST, and in LO-CARB vs HI-CARB (*dz* = 1.00-2.09; all *P* < .01).

### Subjective Exercise Responses

Participants reported lower pre-exercise energy in FAST vs LO-CARB (*dz* = 0.78; *P* = .044) and HI-CARB (*dz* = 0.91; *P* = .028), although motivation, tiredness, and readiness to exercise were not different between trials (*dz* = 0.00-0.58; all *P* ≥ .121). Pre-exercise positive affect (*dz­* = 0.32-0.51; *P* = .103) and negative affect (*­dz* = 0.28-0.66; *P* = .137)*­*, enjoyment of exercise sessions (*dz* = 0.12-0.55; *P ­*= .186), rating of perceived exertion (*dz* = 0.00-1.47*­*; *P* = .070), and heart rate (*dz* = 0.26-0.39; *P* = .249) were not different between trials.

### Meal Perceptions

Overall pleasantness of the lunch meal was lower in LO-CARB (*dz* = 2.18; *P* = .011). The drink was rated as both creamier and thicker in LO-CARB (*dz* = 0.80-2.49; *P* ≤ .014), with no further differences in perceptions (*dz* = 0.32-0.95; *P* ≥ .058). The sandwich was rated as less pleasant and chewier in LO-CARB (*dz* = 1.02-1.16; *P* ≤ .047), with no further perceptual differences (*dz* = 0.42-0.51; *P* ≥ .143).

## Discussion

The novel aspects of the present study were that a low-carbohydrate, high-protein lunch increased fat oxidation during late-afternoon/early-evening exercise compared to an energy-matched high-carbohydrate, lower-protein lunch, while also increasing the secretion of anorexigenic hormones PYY and GLP-1 from the gastrointestinal tract. Accordingly, the LO-CARB lunch suppressed appetite and reduced ad libitum energy intake in the evening by ∼315 kcal compared to HI-CARB, and by ∼385 kcal compared to FAST. These findings suggest that carbohydrate restriction via consuming a low-carbohydrate, high-protein lunch could be used to achieve many of the positive metabolic responses achieved from fasted exercise and mitigate the appetite-related challenges associated with fasted exercise via endocrine signaling.

Our findings showed that fat oxidation was increased by 8.00 ± 3.83 g during 1 hour of exercise performed 3 hours after consuming a low-carbohydrate (0.2 g·kg body mass^−1^ carbohydrate; 6% of energy), high-protein (∼2 g·kg body mass^−1^ protein; ∼53% of energy) lunch, compared with an isocaloric high-carbohydrate (2 g·kg body mass^−1^ carbohydrate; 55% of energy), lower-protein (∼0.4 g·kg body mass^−1^ protein; 10% of energy) lunch, albeit to a lesser extent than after an 8-hour fast (11.66 ± 6.63 g). This builds upon previous observations that similar fat oxidation can be achieved during morning exercise with low-carbohydrate, high-protein feeding or complete fasting (14–16). The ∼65% difference in fat oxidation between protein- and carbohydrate-fed exercise observed in the present study is considerably greater than the ∼19% difference reported in previous work (15). As the inhibition of fat oxidation during fed exercise is governed primarily by the insulinemic response to carbohydrate ingestion (31), this is likely due to the larger carbohydrate content of the LO-CARB meal compared to the previous study. Protein feeding does not appear to attenuate fat oxidation rates to the same extent (∼20% difference in fat oxidation between LO-CARB and fasting), suggesting that substituting pre-exercise carbohydrate for protein may achieve much of the metabolic response associated with increased fat oxidation, without enduring extended fasting during the day.

The stepwise increase in fat oxidation between trials was mirrored by a stepwise reduction in insulin concentrations. Consuming carbohydrate increases plasma glucose and insulin concentrations (32), inhibiting hormone-sensitive lipase activity and lipolysis (33), and stimulating fatty acid re-esterification in adipose tissue (34, 35). This ultimately reduces fatty acid availability for oxidation during exercise after carbohydrate intake (31, 32). Accordingly, plasma NEFA concentrations showed stepwise increases between trials, in line with differences in fat oxidation. The fat content of the pre-exercise meals was closely matched (44 ± 5 g vs 50 ± 6 g, or 34% vs 38% of energy), so it is unlikely that the differences in NEFA concentrations following the LO-CARB and HI-CARB meals were a product of dietary fat appearance, but rather indicate increased mobilization of endogenous lipid stores in LO-CARB.

Plasma glycerol concentrations, which are often used as a surrogate marker of adipose tissue lipolysis (36), were, however, only elevated during FAST. This suggests that different mechanisms may explain the increased fat oxidation in LO-CARB and FAST, likely increased intramuscular triglyceride utilization (32). It should be noted, however, that our data reflect single time-point measures of plasma glycerol concentrations, and so it cannot be distinguished whether changes represent alterations in glycerol appearance (lipolytic rate) or glycerol uptake (37, 38). Therefore, it remains possible that any subtle differences in lipolytic rate between LO-CARB and HI-CARB trials may have been masked by changes in glycerol uptake. Studies have, however, reported elevated NEFA and glycerol concentrations during exercise after smaller doses of protein (14, 39, 40), suggesting the high protein dose and the resultant insulin concentrations in LO-CARB might have reduced lipolysis and fat oxidation compared to FAST. This is supported by observations that even small increases in plasma insulin concentrations can suppress lipolysis (41).

Postprandial concentrations of GLP-1 and PYY were greater in LO-CARB compared to HI-CARB and FAST. This is likely due to the increased protein content of the LO-CARB meal, as evidence supports a dose-dependent relationship between protein intake and postprandial concentrations of GLP-1 and PYY (42, 43). Both GLP-1 and PYY are secreted from intestinal L-cells in response to nutrient ingestion and are associated with reduced appetite and food intake in humans via central effects on the hypothalamus, as well as other mechanisms including the slowing of gastric emptying (44). In addition to its effects on appetite, GLP-1 is an incretin, enhancing glucose-dependent insulin secretion (45), indicating that elevated GLP-1 in LO-CARB also has the potential to benefit postprandial glucose control at subsequent meals. Acylated ghrelin is an orexigenic hormone secreted by the stomach and is often regarded as a biological mechanism to promote hunger and food intake (46). Acylated ghrelin was suppressed by both lunch meals compared to fasting but was not different between LO-CARB and HI-CARB. This suggests that the increase in protein intake in LO-CARB did not alter the acylated ghrelin response, agreeing with some (42, 47), but not all (48) previous studies. However, acylated ghrelin concentrations typically fall rapidly after the onset of food intake, often reaching nadir values within 1 hour (49). Therefore, although our findings indicate the appetite-suppressing effects of the LO-CARB lunch were likely mediated via GLP-1 and PYY, delaying the first blood sample until 1 hour post-lunch may have missed potential differences in acylated ghrelin concentrations between LO-CARB and HI-CARB meals.

The lunch provided during LO-CARB reduced evening energy intake by 22% and 27% compared to HI-CARB and FAST. These responses may have been mediated by increased secretion of GLP-1 and PYY. Our findings corroborate those of Oliveira et al (40), who similarly reported lower post-exercise hunger and greater post-exercise concentrations of PYY and GLP-1 when performed after a high-protein meal. As well as suppressing appetite in the postprandial period, meals with increased protein content have been shown to reduce appetite and promote satiety during the meal (50). It is plausible that the high-protein content of the lunch provided in LO-CARB may have reduced the volume of food consumed at this eating occasion. A smaller meal would generally induce a smaller insulinemic response, possibly allowing for greater rates of fat oxidation during exercise, which may have resulted in closer mirroring of fasted exercise. We chose to match pre-exercise energy intake within the 2 eating occasions in this study, so it was not possible to assess the effect of meal composition on within-meal satiety, but this does represent an interesting avenue for future research. It should be acknowledged that the LO-CARB lunch contained an additional ∼9 g fiber than the HI-CARB lunch. Indeed, dietary fiber can suppress hunger and reduce subsequent energy intake via mechanisms including prolonged oral processing and increased gastric distension (51), which, in addition to its protein content, may have contributed to the superior appetite-suppressing effects of the LO-CARB lunch.

One important factor to note is that the reduction in ad libitum evening energy intake after the LO-CARB meal only compensated for ∼32% of the lunch, so energy intake over the day was still 803 ± 278 kcal lower in FAST. This is consistent with previous studies exploring overnight-fasted morning exercise (3, 4, 9, 10), but it is important to acknowledge the additional challenges associated with fasted exercise later in the day. Slater et al (13) showed that fasting from 11:30 until exercise at 18:30 increased appetite and reduced motivation to exercise, exercise enjoyment, and exercise performance, agreeing with previous findings showing that elevated appetite can reduce resistance exercise performance (52). Similarly, the present study showed that fasting before late-afternoon/early-evening exercise increased appetite and reduced pre-exercise energy levels compared to both LO-CARB and HI-CARB, although other subjective markers including motivation, tiredness, and readiness to exercise were unaffected. In any case, a low-carbohydrate, high-protein lunch may help achieve a better psychological state for engaging in regular exercise, while still increasing fat oxidation.

While this study provides novel insights into the metabolic, perceptual, and energy intake responses to late-afternoon/early-evening exercise after a low-carbohydrate, high-protein lunch, findings must be interpreted in light of the study design. First, rates of substrate oxidation and energy expenditure were not corrected for rates of protein oxidation. Although rates of protein oxidation during exercise are generally considered negligible (26), protein content of the low-carbohydrate lunch was high, so it is possible that the contribution of protein to oxidative metabolism was greater than previously assessed. Secondly, we aimed to compare meals closely matched for taste/texture that contained ecologically valid components typically consumed at lunch in the real world. The resultant high protein content of the low-carbohydrate lunch may be challenging to replicate within the real world, although previous studies have shown increased fat oxidation (14–16) and reduced appetite (40) following smaller, more ecologically valid protein doses. This study also recruited lean, healthy, and active men, meaning that results cannot be directly extrapolated to other population groups, particularly individuals with overweight or obesity, female individuals, or older adults, who may respond differently to fasting-based interventions (13, 53 ). For example, females generally exhibit higher relative rates of fat oxidation during exercise than males (54), potentially enhancing the metabolic benefits associated with increased fat oxidation during fasted or carbohydrate-restricted exercise. In contrast, older adults often have reduced fat oxidative capacity (55), which may attenuate the potential benefits of such exercise interventions. Finally, this was an acute study, so it is not known whether these findings would persist chronically.

## Conclusion

This study showed that the acute consumption of a low-carbohydrate, high-protein lunch before late-afternoon/early-evening exercise (ie, 16:15) increased fat oxidation compared to a high-carbohydrate, lower-protein lunch, although the increase was less than that following an 8 hours fast. The low-carbohydrate, high-protein lunch also increased satiety-related hormone concentrations, and reduced subjective appetite and subsequent energy intake, meaning that this meal composition could offer some of the metabolic benefits associated with fasted exercise without the need to endure daytime fasting. Future studies are required to explore whether acute exercise performed after a low-carbohydrate, high-protein meal can be implemented on a regular basis as a method of managing body weight/composition and maintaining metabolic health.

## Data Availability

Some or all datasets generated during and/or analyzed during the current study are not publicly available but are available from the corresponding author on reasonable request.

## Clinical Trial Information

https://clinicaltrials.gov/ct2/show/NCT05107583

## Abbreviations

ANOVA: analysis of variance
CV: coefficient of variation
DTE: desire to eat
EER: estimated energy requirements
FAST: no lunch (extended fasting) experimental trial
GLP-1: glucagon-like peptide 1
HI-CARB: high-carbohydrate lunch experimental trial
iAUC: incremental area under the curve
LO-CARB: low-carbohydrate lunch experimental trial
LoD: limit of detection
NEFA: nonesterified fatty acids
PFC: prospective food consumption
PYY: peptide YY
tAUC: total area under the curve
VAS: visual analog scale

## References

[1] Pedersen BK, Saltin B. Exercise as medicine—evidence for prescribing exercise as therapy in 26 different chronic diseases. Scand J Med Sci Sports. 2015;25(S3):1‐72. 10.1111/sms.12581 26606383

[2] Donnelly JE, Blair SN, Jakicic JM, Manore MM, Rankin JW, Smith BK. Appropriate physical activity intervention strategies for weight loss and prevention of weight regain for adults. Med Sci Sports Exerc. 2009;41(2):459. 19127177 10.1249/MSS.0b013e3181949333

[3] Edinburgh RM, Hengist A, Smith HA, et al Skipping breakfast before exercise creates a more negative 24-hour energy balance: a randomized controlled trial in healthy physically active young men. J Nutr. 2019;149(8):1326‐1334. 31321428 10.1093/jn/nxz018 PMC6675614

[4] Gonzalez JT, Veasey RC, Rumbold PLS, Stevenson EJ. Breakfast and exercise contingently affect postprandial metabolism and energy balance in physically active males. Br J Nutr. 2013;110(4):721‐732. 23340006 10.1017/S0007114512005582

[5] De Bock K, Derave W, Eijnde BO, et al Effect of training in the fasted state on metabolic responses during exercise with carbohydrate intake. J Appl Physiol. 2008;104(4):1045‐1055. 18276898 10.1152/japplphysiol.01195.2007

[6] Van Proeyen K, Szlufcik K, Nielens H, et al Training in the fasted state improves glucose tolerance during fat-rich diet: fasted training and fat-rich diet. J Physiol. 2010;588(21):4289‐4302. 20837645 10.1113/jphysiol.2010.196493 PMC3002457

[7] Robinson SL, Hattersley J, Frost GS, Chambers ES, Wallis GA. Maximal fat oxidation during exercise is positively associated with 24-hour fat oxidation and insulin sensitivity in young, healthy men. J Appl Physiol. 2015;118(11):1415‐1422. 25814634 10.1152/japplphysiol.00058.2015

[8] Edinburgh RM, Bradley HE, Abdullah N-F, et al Lipid metabolism links nutrient-exercise timing to insulin sensitivity in men classified as overweight or obese. J Clin Endocrinol Metab. 2020;105(3):660‐676. 31628477 10.1210/clinem/dgz104 PMC7112968

[9] Bachman JL, Deitrick RW, Hillman AR. Exercising in the fasted state reduced 24-hour energy intake in active male adults. J Nutr Metab. 2016;2016:1‐7. 10.1155/2016/1984198 PMC5050386 27738523

[10] Griffiths A, Deighton K, Shannon OM, et al Appetite and energy intake responses to breakfast consumption and carbohydrate supplementation in hypoxia. Appetite. 2020;147:104564. 31870935 10.1016/j.appet.2019.104564

[11] Smith HA, Betts JA. Nutrient timing and metabolic regulation. J Physiol. 2022;600(6):1299‐1312. 35038774 10.1113/JP280756 PMC9305539

[12] McIver VJ, Mattin LR, Evans GH, Yau AMW. Diurnal influences of fasted and non-fasted brisk walking on gastric emptying rate, metabolic responses, and appetite in healthy males. Appetite. 2019;143:104411. 31445052 10.1016/j.appet.2019.104411

[13] Slater T, Mode WJA, Pinkney MG, et al Fasting before evening exercise reduces net energy intake and increases fat oxidation, but impairs performance in healthy males and females. Int J Sport Nutr Exerc Metab. 2022;33(1):11‐22. 36170970 10.1123/ijsnem.2022-0132

[14] Impey SG, Smith D, Robinson AL, et al Leucine-enriched protein feeding does not impair exercise-induced free fatty acid availability and lipid oxidation: beneficial implications for training in carbohydrate-restricted states. Amino Acids. 2015;47(2):407‐416. 25471599 10.1007/s00726-014-1876-y

[15] Rothschild JA, Kilding AE, Broome SC, Stewart T, Cronin JB, Plews DJ. Pre-Exercise carbohydrate or protein ingestion influences substrate oxidation but not performance or hunger compared with cycling in the fasted state. Nutrients. 2021;13(4):1291. 33919779 10.3390/nu13041291 PMC8070691

[16] Taylor C, Bartlett JD, van de Graaf CS, et al Protein ingestion does not impair exercise-induced AMPK signalling when in a glycogen-depleted state: implications for train-low compete-high. Eur J Appl Physiol. 2013;113(6):1457‐1468. 23263742 10.1007/s00421-012-2574-7

[17] Aird TP, Farquharson AJ, Bermingham KM , et al Divergent serum metabolomic, skeletal muscle signaling, transcriptomic, and performance adaptations to fasted versus whey protein-fed sprint interval training. Am J Physiol Endocrinol Metab. 2021;321(6):E802‐E820. 34747202 10.1152/ajpendo.00265.2021 PMC8906818

[18] Larsen MS, Holm L, Svart MV, et al Effects of protein intake prior to carbohydrate-restricted endurance exercise: a randomized crossover trial. J Int Soc Sports Nutr. 2020;17(1):7. 31992300 10.1186/s12970-020-0338-z PMC6986159

[19] Rolls BJ, Hetherington M, Burley VJ. The specificity of satiety: the influence of foods of different macronutrient content on the development of satiety. Physiol Behav. 1988;43(2):145‐153. 3212049 10.1016/0031-9384(88)90230-2

[20] Stunkard AJ, Messick S. The three-factor eating questionnaire to measure dietary restraint, disinhibition and hunger. J Psychosom Res. 1985;29(1):71‐83. 3981480 10.1016/0022-3999(85)90010-8

[21] Hill JO, Peters JC, Wyatt HR. Using the energy gap to address obesity: a commentary. J Am Diet Assoc. 2009;109(11):1848‐1853. 19857625 10.1016/j.jada.2009.08.007 PMC2796109

[22] Durnin JVGA, Womersley J. Body fat assessed from total body density and its estimation from skinfold thickness: measurements on 481 men and women aged from 16 to 72 years. Br J Nutr. 1974;32(1):77‐97. 4843734 10.1079/bjn19740060

[23] Mifflin M, St Jeor S, Hill L, Scott B, Daugherty S, Koh Y. A new predictive equation for resting energy expenditure in healthy individuals. Am J Clin Nutr. 1990;51(2):241‐247. 2305711 10.1093/ajcn/51.2.241

[24] Slater T, Mode WJA, Bonnard LC, et al Supplemental tables for: Substituting carbohydrate at lunch for added protein increases fat oxidation during subsequent exercise in healthy males. Zenodo. Accessed 23 April 23, 2024. 10.5281/zenodo.11045689 PMC11834723 38609167

[25] Griffiths A, Humphreys S, Clark M, Fielding B, Frayn K. Immediate metabolic availability of dietary fat in combination with carbohydrate. Am J Clin Nutr. 1994;59(1):53‐59. 8279403 10.1093/ajcn/59.1.53

[26] Jeukendrup AE, Wallis GA. Measurement of substrate oxidation during exercise by means of gas exchange measurements. Int J Sports Med. 2005;26(S1):S28‐S37. 15702454 10.1055/s-2004-830512

[27] Watson D, Clark LA, Tellegen A. Development and validation of brief measures of positive and negative affect: the PANAS scales. J Pers Soc Psychol. 1988;54(6):1063‐1070. 3397865 10.1037//0022-3514.54.6.1063

[28] Raedeke TD . The relationship between enjoyment and affective responses to exercise. J Appl Sport Psychol. 2007;19(1):105‐115.

[29] Narang BJ, Atkinson G, Gonzalez JT, Betts JA. A tool to explore discrete-time data: the time series response analyser. Int J Sport Nutr Exerc Metab. 2020;30(5):374‐381. 32726749 10.1123/ijsnem.2020-0150

[30] Cohen J. Statistical Power Analysis for the Behavioral Sciences. 2nd ed. Routledge Academic; 1988.

[31] Vieira AF, Costa RR, Macedo RCO, Coconcelli L, Kruel LFM. Effects of aerobic exercise performed in fasted v. fed state on fat and carbohydrate metabolism in adults: a systematic review and meta-analysis. Br J Nutr. 2016;116(7):1153‐1164. 27609363 10.1017/S0007114516003160

[32] Coyle EF, Jeukendrup AE, Wagenmakers AJ, Saris WH. Fatty acid oxidation is directly regulated by carbohydrate metabolism during exercise. Am J Physiol Endocrinol Metab. 1997;273(2):E268‐E275. 10.1152/ajpendo.1997.273.2.E268 9277379

[33] Saltiel AR, Kahn CR. Insulin signalling and the regulation of glucose and lipid metabolism. Nature. 2001;414(6865):799‐806. 11742412 10.1038/414799a

[34] Enevoldsen LH, Simonsen L, Macdonald IA, Bülow J. The combined effects of exercise and food intake on adipose tissue and splanchnic metabolism. J Physiol. 2004;561(3):871‐882. 15498802 10.1113/jphysiol.2004.076588 PMC1665376

[35] Frayn KN, Coppack SW, Fielding BA, Humphreys SM. Coordinated regulation of hormone-sensitive lipase and lipoprotein lipase in human adipose tissue in vivo: Implications for the control of fat storage and fat mobilization. Adv Enzyme Regul. 1995;35:163‐178. 7572342 10.1016/0065-2571(94)00011-q

[36] Robinson S, Chambers E, Fletcher G, Wallis G. Lipolytic markers, insulin and resting fat oxidation are associated with maximal fat oxidation. Int J Sports Med. 2016;37(08):607‐613. 27116342 10.1055/s-0042-100291

[37] van Loon LJC, Koopman R, Stegen JHCH, Wagenmakers AJM, Keizer HA, Saris WHM. Intramyocellular lipids form an important substrate source during moderate intensity exercise in endurance‐trained males in a fasted state. J Physiol. 2003;553(2):611‐625. 14514877 10.1113/jphysiol.2003.052431 PMC2343576

[38] van Hall G, Sacchetti M, Rådegran G, Saltin B. Human skeletal muscle fatty acid and glycerol metabolism during rest, exercise and recovery. J Physiol. 2002;543(3):1047‐1058. 12231658 10.1113/jphysiol.2002.023796 PMC2290548

[39] Erdmann J, Tholl S, Schusdziarra V. Effect of carbohydrate- and protein-rich meals on exercise-induced activation of lipolysis in obese subjects. Horm Metab Res. 2010;42(04):290‐294. 20094973 10.1055/s-0029-1243637

[40] Oliveira CLP, Boulé NG, Berg A, Sharma AM, Elliott SA, Siervo M, et al Consumption of a high-protein meal replacement leads to higher fat oxidation, suppression of hunger, and improved metabolic profile after an exercise session. Nutrients. 2021;13(1):155. 33466462 10.3390/nu13010155 PMC7824960

[41] Bonadonna RC, Leif G, Kraemer N, Ferrannini E, Prato SD, DeFronzo RA. Obesity and insulin resistance in humans: A dose-response study. Metabolism. 1990;39(5):452‐459. 2186255 10.1016/0026-0495(90)90002-t

[42] Belza A, Ritz C, Sørensen M, Holst JJ, Rehfeld JF, Astrup A. Contribution of gastroenteropancreatic appetite hormones to protein-induced satiety. Am J Clin Nutr. 2013;97(5):980‐989. 23466396 10.3945/ajcn.112.047563

[43] Batterham RL, Heffron H, Kapoor S, et al Critical role for peptide YY in protein-mediated satiation and body-weight regulation. Cell Metab. 2006;4(3):223‐233. 16950139 10.1016/j.cmet.2006.08.001

[44] Suzuki K, Simpson KA, Minnion JS, Shillito JC, Bloom SR. The role of gut hormones and the hypothalamus in appetite regulation. Endocr J. 2010;57(5):359‐372. 20424341 10.1507/endocrj.k10e-077

[45] Watkins JD, Koumanov F, Gonzalez JT. Protein- and calcium- mediated GLP-1 secretion: a narrative review. Adv Nutr. 2021;12(6):2540‐2552. 34192748 10.1093/advances/nmab078 PMC8634310

[46] Cummings DE, Frayo RS, Marmonier C, Aubert R, Chapelot D. Plasma ghrelin levels and hunger scores in humans initiating meals voluntarily without time- and food-related cues. Am J Physiol Endocrinol Metab. 2004;287(2):E297‐E304. 15039149 10.1152/ajpendo.00582.2003

[47] Erdmann J, Töpsch R, Lippl F, Gussmann P, Schusdziarra V. Postprandial response of plasma ghrelin levels to various test meals in relation to food intake, plasma insulin, and glucose. J Clin Endocrinol Metab. 2004;89(6):3048‐3054. 15181097 10.1210/jc.2003-031610

[48] Blom WAM, Lluch A, Stafleu A, Vinoy S, Holst JJ, Schaafsma G, et al Effect of a high-protein breakfast on the postprandial ghrelin response. Am J Clin Nutr. 2006;83(2):211‐220. 16469977 10.1093/ajcn/83.2.211

[49] Callahan HS, Cummings DE, Pepe MS, Breen PA, Matthys CC, Weigle DS. Postprandial suppression of plasma ghrelin level is proportional to ingested caloric load but does not predict intermeal interval in humans. J Clin Endocrinol Metab. 2004;89(3):1319‐1324. 15001628 10.1210/jc.2003-031267

[50] Westerterp-Plantenga MS, Rolland V, Wilson SAJ, Westerterp KR. Satiety related to 24 h diet-induced thermogenesis during high protein/carbohydrate vs high fat diets measured in a respiration chamber. Eur J Clin Nutr. 1999;53(6):495‐502. 10403587 10.1038/sj.ejcn.1600782

[51] Slavin J, Green H. Dietary fibre and satiety. Nutr Bull. 2007;32(s1):32‐42.

[52] Naharudin MN, Yusof A, Clayton DJ, James LJ. Starving your performance? reduced preexercise hunger increases resistance exercise performance. Int J Sports Physiol Perform. 2022;17(3):458‐464. 34872065 10.1123/ijspp.2021-0166

[53] Gonzalez JT, Richardson JD, Chowdhury EA, et al Molecular adaptations of adipose tissue to 6 weeks of morning fasting vs. daily breakfast consumption in lean and obese adults. J Physiol. 2018;596(4):609‐622. 29193093 10.1113/JP275113 PMC5813615

[54] Chrzanowski‐Smith OJ, Edinburgh RM, Smith E, et al Resting skeletal muscle PNPLA2 (ATGL) and CPT1B are associated with peak fat oxidation rates in men and women but do not explain observed sex differences. Exp Physiol. 2021;106(5):1208‐1223. 33675111 10.1113/EP089431

[55] Sial S, Coggan AR, Carroll R, Goodwin J, Klein S. Fat and carbohydrate metabolism during exercise in elderly and young subjects. Am J Physiol. 1996;271(6):E983‐E989. 8997215 10.1152/ajpendo.1996.271.6.E983

